
JOURNAL INFORMATION
==============================
NLM Title Abbreviation: Innov Surg Sci
Journal ID: Innov Surg Sci
NLM Journal ID: 101708165
Journal ID: iss

Innovative Surgical Sciences

EISSN: 2364-7485
Publisher: De Gruyter

ARTICLE INFORMATION
==============================
PMCID: PMC11451810
DOI: 10.1515/iss-2024-2007
Article ID: iss-2024-2007
Article version: 1
Subjects: Article

Abstracts Interdisciplinary

Electronic publication date: 2024 Apr 9
Publication date: 2024 Apr
Volume: 9
Issue: Suppl1
First page: 280
Last page: 302
Copyright: © 2024 by Walter de Gruyter Berlin/Boston
Copyright year: 2024
Copyright holder: De Gruyter
License: This work is licensed under the Creative Commons Attribution 4.0 International License.
License URL: https://creativecommons.org/licenses/by/4.0/

Page count: 23

==============================

Abstracts – DCK 2024 – 141st Congress of the German Society of Surgery – Leipzig, April 24–26 • DOI 10.1515/iss-2024-2007
Innov Surg Sci 2024; 9 (Special Suppl 1): s280–s302

### Are we able to delineate curative treatment approaches in cases of TTC7A-deficiency? Case Report and Review of the literature

ID: 73

Hans Christian Schmidt[1], Julia Imhoff[2], Konrad Reinshagen[1], Christian Tomuschat[1]

[1]*University Medical Center Hamburg-Eppendorf, Department of Pediatric Surgery*, [2]*University Medical Center Hamburg-Eppendorf*

**Background:**

TTC7A-deficiency is a rare hereditary condition characterized by combined immunodeficiency with multiple intestinal atresias presenting in heterogenous degrees of severity [1] and is drawn back to consanguinity [2]. The genetic disorder leads to disrupted and inverse construction of the apicobasal polarity in enterocytes as shown in intestinal organoids, derived from TTC7A-patients [3]. The defect is also associated with a very early onset of inflammatory bowel disease [4]. Immunodeficiency can be explained by inappropriate activation of the Rho-kinase and impairment of the actin cytoskeleton dynamic, resulting in modified proliferation, adhesion, and migratory capacities of the lymphocytes [5]. The prognosis is fatal in approximately two thirds of the cases and treatment is based on palliative concepts [6, 7]. Treatment options are limited, e.g., intestinal transplantation, as patients with multiple atresia and immunodeficiency are predisposed to develop severe Graft vs. Host disease [8]. Therefore, unravelling of new signaling pathways in the pathogenesis are of great importance to optimize the use of remaining therapeutic options. In vitro treatment by pharmacological inhibition of the Rho-kinase has shown to be effective [3]. The objective of this case report is to highlight treatment difficulties in the genetic disorder of TTC7A-deficiency and to review the recent understanding of the pathogenesis.

**Materials and methods:**

A male patient with prenatally suspected intestinal atresia underwent comprehensive assessment. The interdisciplinary evaluation included departments of human genetics, immunology, cardiology, neurology, radiology, pathology, neonatal intensive care medicine, and pediatric surgery at the University Medical Center Hamburg-Eppendorf. Literature was reviewed regarding the recent understanding of pathogenesis and treatment opportunities in patients with TTC7A-deficiency.

**Results:**

The study involved a male neonate with prepartal suspicion of intestinal atresia, characterized by a singular gastric bubble and an otherwise airless abdomen. Genomic analysis confirmed a homozygous TTC7A-deficiency (Q82.8 Combined Immunodeficiency-Enteropathy Spectrum). Born spontaneously at 37+2 weeks, he was immediately admitted to the neonatal intensive care unit. Both sonographic and X-ray examinations corroborated the suspicion of intestinal atresia, with the presence of a singular gastric bubble and a mostly airless abdomen.

Following his birth, the patient received treatment involving gastric tube placement and total parenteral nutrition. Cerebral sonography showed no abnormalities, but echocardiography revealed a narrow aortic arch, a patent foramen ovale, and a patent ductus arteriosus. Immunological analysis indicated cytotoxic T-cell deficiency, reduced memory B-cells, and a disproportionate reduction in regulatory T-cells during T-cell differentiation. Surgical intervention was performed on the second day of life with the goal of restoring intestinal continuity. An exploratory laparotomy uncovered multiple intestinal atresias, totaling > 30, which included pyloric atresia, duodenal atresia, multiple jejunal and ileal atresias, and colonic atresia. Surgical procedures included pylorotomy and duodeno-jejunostomy, jejuno-jejunal bypass, Meckel's diverticulum resection, and creation of a descendostomy. Histological examination of Meckel's diverticulum showed inflammatory changes, mucosal edema, hemorrhages, but no dysplasia or malignancy.

Although gastric distension improved postoperatively, the gastric tube still drained significant amounts, and there was a lack of peristalsis. Consequently, on the 16th day of life, a second laparotomy was performed, which involved scarred gastroduodenostomy dilatation, pylorus re-bypass anastomosis, jejunal re-anastomosis via jejuno-jejunostomy, and the creation of a double-barreled jejunostoma.

Open Access. © 2024 The Author(s), published by De Gruyter. This work is licensed under the Creative Commons Attribution 4.0 International License.

Abstracts – DCK 2024 – 141st Congress of the German Society of Surgery – Leipzig, April 24–26 • DOI 10.1515/iss-2024-2007
Innov Surg Sci 2024; 9 (Special Suppl 1): s280–s302

Histological examination of removed intestinal tissue revealed erosions, ulcerations, disrupted mucosal architecture, foreign body giant cells, submucosal edema, and regular ganglions with Cajal cell presence. Given ongoing poor growth and ongoing discontinuity, a tunneled central venous catheter (Broviac) was implanted in the left internal jugular vein at 50 days of age, initiating a palliative therapeutic approach.

**Conclusion:**
Despite interdisciplinary high-end treatment, medical care of complex genetic disorders, such as TTC7A-deficiency remain challenging. Future therapeutics including inhibitors of Rho-kinase seem promising [3], but for now delineation of curative concepts remain elusive as so far no treatment exist that can maintain clinical remission in TTC7A patients [6].

**References**
1. Chen, R. et al. Whole-exome sequencing identifies tetratricopeptide repeat domain 7A (TTC7A) mutations for combined immunodeficiency with intestinal atresias. *J Allergy Clin Immunol* **132,** 656-664.e17 (2013).
2. Guttman, F. M. et al. Multiple atresias and a new syndrome of hereditary multiple atresias involving the gastrointestinal tract from stomach to rectum. *J Pediatr Surg* **8,** 633-40 (1973).
3. Bigorgne, A. E. et al. TTC7A mutations disrupt intestinal epithelial apicobasal polarity. *J Clin Invest* **124,** 328-37 (2014).
4. Avitzur, Y. et al. Mutations in tetratricopeptide repeat domain 7A result in a severe form of very early onset inflammatory bowel disease. *Gastroenterology* **146,** 1028-39 (2014).
5. Lemoine, R. et al. Immune deficiency-related enteropathy-lymphocytopenia-alopecia syndrome results from tetratricopeptide repeat domain 7A deficiency. *J Allergy Clin Immunol* **134,** 1354-1364.e6 (2014).
6. Jardine, S., Dhingani, N. & Muise, A. M. TTC7A: Steward of Intestinal Health. *Cell Mol Gastroenterol Hepatol* **7,** 555-570 (2019).
7. Samuels, M. E. et al. Exome sequencing identifies mutations in the gene TTC7A in French-Canadian cases with hereditary multiple intestinal atresia. *J Med Genet* **50,** 324-9 (2013).
8. Fischer, R. T. et al. Intestinal transplantation in children with multiple intestinal atresias and immunodeficiency. *Pediatr Transplant* **18,** 190-6 (2014).

**Neurosurgery for brain metastases: From an “end-of-life” situation to a hallmark of interdisciplinary treatment**
ID: 813

Stephanie T. Jünger[1 2], Maximilian I. Ruge[2 3], Daniel Ruess[2 3], Jürgen Hampl[1 2], Roland Goldbrunner[1 2], Stefan Grau[4]

[1]*Center for Neurosurgery, Department of General Neurosurgery, Faculty of Medicine and University Hospital Cologne, University of Cologne, Cologne, Germany*, [2]*Center for Integrated Oncology, Faculty of Medicine and University Hospital Cologne, University of Cologne, Cologne, Germany*, [3]*Center for Neurosurgery, Department of Stereotactic and Functional Neurosurgery, Faculty of Medicine and University Hospital Cologne, University of Cologne, Cologne, Germany*, [4]*Department of Neurosurgery, Klinikum Fulda, Academic Hospital of University of Marburg, Marburg, Germany.*

**Background:**
To asses demographic, clinical and treatment-related parameters as well as the course of disease including local progression and overall survival in patients undergoing surgical resection for brain metastases (BM).

**Materials and methods:**
We retrospectively identified patients >18 years, who underwent surgery after initial BM diagnosis at our centre between 2012 and 2022. We retrieved bbaseline clinical and treatment-related parameters and calculated post-treatment overall and event-free survival using Kaplan-Meyer analysis. Prognostic factors were identified using multivariate Cox-regression.

**Results:**
We included 604 patients were included. Gender distribution was almost balanced with 52.2% female and 47.8% male patients. Primary tumors (PT) comprised NSCLC (n=266, 44.0%), SCLC (n=23, 3.8%), breast cancer (n=86, 14.2%), melanoma (n=61, 10.1%), colorectal carcinoma (n=41, 6.8%), esophagus carcinoma (n=23, 3.8%), kidney cancer (n=16, 2.6%), prostate carcinoma (n=13, 2.2%), urothelial carcinoma (n=15, 2.5%), CUP-syndrome (n=21, 3.5%) and others (n=39, 6.5%).
Seventy-five patients had one or more additional tumors.
Regarding treatment for PT, 48.2% underwent surgery, 41.1% received chemotherapy, 26.7% radiotherapy, 8.1% immunotherapy, 19.5% targeted and 35.4% no therapy before initial BM diagnosis.
While PT was diagnosed at a mean age of 59.9 years (range: 5-87), mean age at initial BM was 62.4 years (range: 19-87).
BM were diagnosed after PT within a mean period of 40 months (range:0-458), 32.8% of BM were diagnosed before the PT, 7.5% within 3 months of PT diagnoses and 59.8% more than 3 months after PT diagnosis.
Almost all patients had symptoms related to their BM at diagnosis. Most patients had one (60.9%) BM, while 24.7% had 2-3, and 14.4% of patients had >3 BM. In 56 patients, systemic disease status was unknown; of the remaining cohort, 170 had stable systemic disease at time of BM diagnosis.
Most patients underwent operation for one BM (90.2%), while 8.9% and 0.8% had two, respectively 3 BM resected.
Karnofsky performance index (KPI) could be improved from a preoperative mean of 75 (20-100) to a postoperative mean of 80 (0-100). Two-hundred-fifty-eight patients clinically improved while 44 deteriorated.
Taking surgical as well as non-surgical complications into account, 120 patients suffered postoperative complications ranging from small wound healing problems to death within 30days (n=7). Two patients had to undergo second surgery for residual tumor.
Most patients received postoperative radiotherapy (84.1%), either WBRT (22.7%) or local radiotherapy including focal radiotherapy (60.1%), neo-adjuvant or postoperative radiosurgery (1.8% and 8.8% respectively). Furthermore, 46.9% received systemic therapy, including targeted therapy in 18.1% and immunotherapy in 18.2%.

Sixty-seven (11.1%) did neither receive radiation nor systemic therapy after BM resection.
For 577 patients, postoperative referral at least for radiation (and mostly oncology as well) was organized at the date of discharge or transfer.
At the time of analysis, 244 patients suffered had intracranial relapse diagnosed on cMRI; relapse was local in 7.9%, local and distant in 9.3%, distant in 13.7%, and 9.4% of patients had leptomeningeal disease.
Forty-five patients were diagnosed with more than one intracranial relapse.
After a mean overall survival of15.3 months (range: 0-131), 344 patients had died at the time of analysis. In 201 patients cause of death was known; 115 patients died from systemic and 72 from intracranial progression, while the remaining 14 patient died from other causes.
Regarding long-term survival, 6.3% of patients survived longer than 5, 12.4% longer than 3, and 18.4% longer than 2 years after initial BM diagnosis.
Univariate survival analysis local radiotherapy showed significant local control for the operated and irradiated BM (p=0.003).
Regarding overall survival gender (p=0.03), age >65 (p=0.007), stable systemic disease (p<0.001), postoperative KPI >70 (p<0.001), radiotherapy (p<0.001), systemic therapy (p<0.001), complications (p=0.007) were significant prognostic factors. In multivariate cox regression analysis stable systemic disease (p<0.001), systemic therapy (p<0.001), postoperative radiotherapy (p<0.001), and postoperative complications (p=0.003) remained independent predictors of survival.

**Conclusion:**
Within the last decades, neurosurgical resection for singular and even in case of more than one BM has increased in frequency.
Our data suggest that surgery may improve the clinical status of the patient and hereby enables adjuvant radiation and systemic treatment, which besides the systemic status remain the strongest predictors of outcome in this patient cohort. However, the impact of systemic disease status should not be underestimated and preoperative staging is inevitable to evaluate whether a patient is suitable for neurosurgical resection. Especially, since the rate of surgical complications in this multi-morbid patient cohort is rather high.
To conclude, our data suggest that neurosurgical resection represents an important aspect of interdisciplinary treatment in patients with BM from systemic cancer, however it emphasizes that resection as a standalone treatment is not useful and that dedicated patient referral to adjuvant treatment is crucial to ensure an optimal outcome.

Abstracts – DCK 2024 – 141st Congress of the German Society of Surgery – Leipzig, April 24–26 • DOI 10.1515/iss-2024-2007
Innov Surg Sci 2024; 9 (Special Suppl 1): s280–s302

**Scalp reconstruction with locoregional and free flaps – a retrospective cohort study**
ID: 230

Olimpiu Bota[1], Tarek Juratli[2], Adrian Dragu[2], Kevin Bienger[2]

[1]*Cluj-Napoca, County Clinical Emergency Hospital, Cluj-Napoca, Romania*, [2]*Dresden, Uniklinikum Dresden, Dresden, Deutschland*

**Background:**
Scalp defect reconstruction requires interdisciplinary cooperation to restore soft tissue and osseous defects. While wound closure and form restoration, often a short-term treatment goal, ensures patient survival, the long-term preservation of the head and neck's integrity and aesthetics is crucial for maintaining quality of life. This study aims to compare, quantify, and establish a safe and reproducible approach to various reconstruction options and the postoperative complication profile for individual scalp defect areas.

**Materials and methods:**
We retrospectively evaluated patients who underwent scalp reconstruction at our institution between March 2017 and April 2022. The inclusion criterion was the presence of a soft tissue defect at the cranium level.

**Results:**
We included 31 patients in the study (17 males, 14 females), with an average age of 61 years (range 17-92 years). Eight patients had received radiotherapy in the affected region. The mean defect size was 72.5 ± 116 cm2 (range 2-441 cm2), and an average of 3 ±2 surgeries had been performed before the plastic surgical treatment was initiated. Eleven patients had only a soft tissue defect, while 20 patients had an associated bone defect. Fifteen of these patients received a cranioplasty. The rotation flap was the most frequently used (n=23), with or without split-thickness skin grafting, followed by the free latissimus dorsi muscle flap with split-thickness skin grafting (n=5), and the free lateral arm flap (n=2). Revision surgeries were necessary in 38.7% of cases due to wound healing disorders (n=9), bleeding (n=2), and cerebrospinal fluid leaks (n=1). Eventually, all wounds were successfully closed.

**Conclusion:**
Complex scalp defects can be closed using local flaps, thereby restoring aesthetics and tissue integrity. Free flaps remain a reliable solution for extensive defects. Moreover, in cases requiring cranioplasty, careful preoperative planning and an uncontaminated wound are essential for successful treatment.

### Detection of mandibular fractures on panoramic radiographs using deep learning

ID: 885

Shankeeth Vinayahalingam[1], Niels van Nistelrooij[1], Bram van Ginneken[1], Daniel Tröltzsch[2], Max Heiland[2], Tabea Flügge[2], Robert Gaudin[2 3]

[1]*Radboud-Universität Nijmegen, Nijmegen, Netherlands*, [2]*Charité Campus Benjamin Franklin, Berlin, Deutschland*, [3]*BIH - Zentrum Digitale Gesundheit, Berlin, Deutschland*

**Background:**

Mandibular fractures are among the most frequent facial traumas in oral and maxillofacial surgery, accounting for 57% of cases. An accurate diagnosis and appropriate treatment plan are vital in achieving optimal re-establishment of occlusion, function and facial aesthetics. This study aims to detect mandibular fractures on panoramic radiographs (PR) automatically.

**Materials and methods:**

1624 PR with fractures were manually annotated and labelled as a reference. A deep learning approach based on Faster R-CNN and Swin-Transformer was trained and validated on 1640 PR with and without fractures. Subsequently, the trained algorithm was applied to a test set consisting of 149 PR with and 171 PR without fractures. The detection accuracy and the area-under-the-curve (AUC) were calculated.

**Results:**

The model achieved a precision of 0.935, recall of 0.960 and F1-score of 0.947. The AUC and AP were 0.977 and 0.963, respectively.

**Conclusion:**

Deep learning-based assistance of clinicians may reduce the misdiagnosis and hence the severe complications

Abstracts – DCK 2024 – 141st Congress of the German Society of Surgery – Leipzig, April 24–26 • DOI 10.1515/iss-2024-2007
Innov Surg Sci 2024; 9 (Special Suppl 1): s280–s302

**Novel Incision for the Treatment of Popliteal Artery Entrapment Syndrome Reduces Postoperative Wound Complication Rate**
ID: 293

Toni Engmann[1], Stephen Stearns[1], Raffaela Faulhaber[1], Merle Göben[1], Stephanie Francalancia[1], Lyle Micheli[2], Joseph Upton[3], Arriyan Dowlatshahi[1]

[1]*Beth Israel Deaconess Medical Center, Department of Orthopaedic Surgery, Boston, United States*, [2]*Boston Children's Hospital, Orthopaedic Surgery, Boston, United States*, [3]*Upton Joseph MD, -, Brookline, United States*

**Background:**
Functional popliteal artery entrapment syndrome (PAES) is a rare condition that leads to compression of the popliteal artery through muscles or ligaments. Surgical release is an established treatment option that consists of debulking the muscles and removing any structures that might compress the artery. We introduce a novel Bruner-type incision for surgical release, which shows fewer postoperative complications than the conventional lazy S incision.

**Materials and methods:**
Patients treated surgically for PAES at our Institution between 1990 and 2022 were identified. Demographic and clinical characteristics, surgical treatment, and outcome variables were assessed. Cases with the conventional lazy S-incision posterior approach and cases with the novel posterior Bruner incision were differentiated. Statistical analysis of the outcomes as well as subgroup analysis was with significance determined at $p < 0.05$.

**Results:**
Fifty patients surgically treated for PAES were identified at our institution. The Bruner approach (14 patients) resulted in a significantly lower rate of complications than the Lazy S approach(36 patients) (29% vs. 67%, p = 0.0253, respectively). Specifically, the rate of wound dehiscence was significantly lower in the Bruner approach group than in the lazy S group (0% vs. 39%, p = 0.0049).

**Conclusion:**
A Bruner-type incision may represent be a better alternative to the lazy S approach to the popliteal fossa. This shows equivalent, if not superior, outcomes with a lower complication rate that merit further investigation.

**Differences between post-bariatric patients with regard to body contouring procedures – explanations and implications**
ID: 11

Britta Pehlke[1 2 3], Lena Busch[1 2 3], Philipp Wiebringhaus[1 2 3], Maximilian Kueckelhaus[1 2 3], Fabian Nehls[1 2 3], Tobias Hirsch[1 2 3], Alexander Dermietzel[1 2 3]

[1]*University Hospital Muenster, Division for Plastic Surgery, Department of Trauma, Hand and Reconstructive Surgery, Münster, Deutschland*, [2]*University Muenster, Department for Plastic and Reconstructive Surgery, Institute for Muskuloskeletal Medicine, Münster, Deutschland*, [3]*Fachklinik Hornheide, Plastic, Reconstructive, and Aesthetic Surgery, Hand Surgery, Münster, Deutschland*

**Background:**
The prevalence of obesity has tripled since 1975. 12% of the world-wide adult population are estimated to be affected by obesity (1) with numbers expected to continue to increase further in upcoming years. Likewise, the number of people undergoing weight loss surgery increases. Although patients experience an improvement of physical and mental health (2) as well as a better quality of life (3), they report impairments of well-being due to excess skin after massive weight loss (4). Body contouring procedures (BCP) lead to a reduction of physical and psychological complaints (5) as well as an improved quality of life and social functioning (6). Prior research found desire for BCP to be as high as 73% for post-bariatric patients (7). However, the rate of patients eventually undergoing BCP is significantly lower with numbers ranging from 7% to 25% depending on country of origin and demographics (8, 9, 10).
The present study aims at describing differences between post-bariatric patients who have undergone BCP, patients who are seeking BCP, patients not seeking BCP and those who are unsure about BCP.

**Materials and methods:**
In a nonrandomized, cross-sectional study, an online survey was distributed to post-bariatric patients through obesity organizations, obesity self-help groups, hospitals, obesity centers and through obesity- and bariatric surgery-related groups on social media platforms. Inclusion criteria were age over 18 years old and a minimum of two years after bariatric surgery. Informed consent was given by all participants. Sociodemographic characteristics were recorded as well as time since bariatric surgery, weight loss and current body mass index. Patients were divided into four groups according to whether they had already undergone BCP, planned to undergo BCP, did not seek BCP or were unsure in this regard.

**Results:**
345 participants fulfilled the inclusion criteria and were included in the study. 40.90% of the participants had already undergone BCP. 36.50% strove for BCP and 7.50% of the patients did not want to undergo BCP. 15.10% were unsure about seeking BCP (figure 1).

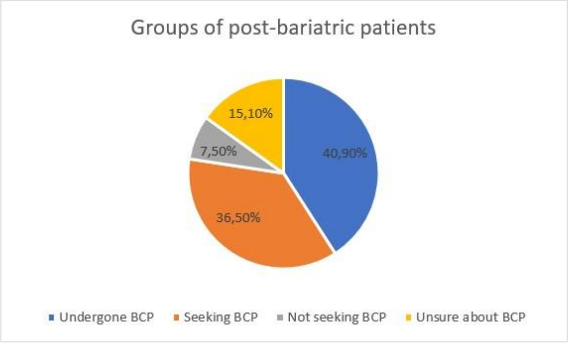

Figure 1. Groups of post-bariatric patients

Patients who had undergone BCP had lost more weight ($F(3, 330) = 7.369$, $p < .001$), had a lower body mass index ($F(3, 332) = 6.080$, $p < .001$) and were younger ($F(3, 341) = 4.661$, $p = .003$) than patients who had not undergone BCP (yet). No difference was found with regard to the time since bariatric surgery. Furthermore, the groups did not differ with regard to marital status or level of education. Women and men were equally represented among the four groups.

85.1 % of patients who had undergone BCP had the surgery covered by their health insurance. Only 7.1 % of the patients paid the treatment for themselves.

| | Age, years | Time since (first) bariatric surgery, months | Weight loss after bariatric surgery, kilograms | Current body-mass index, kg/ m² |
|---|---|---|---|---|
| Patients who have undergone BCP | 44.65±8.93 | 71.60±50.70 | 72.14±22.00 | 30.41±5.75 |
| Patients seeking BCP | 42.48±9.44 | 55.17±49.65 | 62.69±23.03 | 32.64±7.61 |
| Patients not seeking BCP | 49.27±10.74 | 60.10±41.66 | 55.04±20.08 | 33.86±9.19 |
| Patients unsure about BCP | 46.00±9.49 | 60.71±45.27 | 61.10±21.66 | 35.07±8.80 |
| Total | 44.41±9.49 | 63.09±49.25 | 65.78±22.81 | 32.20±7.42 |

Table 1. Patient characteristics by subgroup of post-bariatric patients

**Conclusion:**
Several differences were found between post-bariatric patients who had already undergone BCP and those who had not undergone BCP yet. A high weight loss, a low current body mass index and a young age seem to be associated with undergoing BCP. This association might be reciprocal with a lower body mass index making BPC more probable and BCP facilitating weight loss maintenance. This has presumably to do with the patients' improved physical and psychological constitution after BCP.
Only a very small proportion of post-bariatric patients does not want to undergo BCP. This indicates that BPC have to be regarded as a substantial aspect of obesity treatment and must be incorporated into a multidisciplinary approach to treat people with obesity. Massive weight loss as experienced after bariatric surgery cannot be concluded suitably without taking into account potentially following BCPs.
The fact that almost all conducted BCP were paid for by the patients' health insurance companies can be an explanation for the discrepancy found between the amount of post-bariatric patients wishing for BCP and the small proportion eventually undergoing BCP. Probably most patients cannot afford to pay for BCP themselves. This might be the reason for the relatively high proportion of patients being unsure about BCP. It is thus important to evaluate medical appropriateness and possibilities as early as possible after or even prior to bariatric surgery and assisting patients in the process of applying for cost coverage. Incorporating reconstructive surgery into obesity centres enables surgeons to inform patients about possibilities and realistic results but also about limits of BCP.

**References:**
1. Abarca-Gómez, L et al. Worldwide trends in body-mass index, underweight, overweight, and obesity from 1975 to 2016: a pooled analysis of 2416 population-based measurement studies in 128.9 million children, adolescents, and adults. Lancet. 2017; 390:2627–2642.

2. de Zwaan M, Enderle J, Wagner S, et al. Anxiety and depression in bariatric surgery patients: a prospective, follow-up study using structured clinical interviews. J Affect Disord. 2011;133(1-2):61-68.

3. Major P, Matłok M, Pędziwiatr M, et al. Quality of Life After Bariatric Surgery. Obes Surg. 2015;25(9):1703-1710.

4. Staalesen T, Fagevik Olsén M, Elander A. Experience of excess skin and desire for body contouring surgery in post-bariatric patients. Obes Surg. 2013;23(10):1632-1644.

5. Nielsen C, Elander A, Staalesen T, Al Nouh M, Fagevik Olsén M. Depressive symptoms before and after abdominoplasty among post-bariatric patients - a cohort study. J Plast Surg Hand Surg. 2022;56(6):381-386.

6. Jiang Z, Zhang G, Huang J, et al. A systematic review of body contouring surgery in post-bariatric patients to determine its prevalence, effects on quality of life, desire, and barriers. Obes Rev. 2021;22(5):e13201.

7. Staalesen T, Olbers T, Dahlgren J, et al. Development of excess skin and request for body-contouring surgery in postbariatric adolescents. Plast Reconstr Surg. 2014;134(4):627-636.

8. Al-Hadithy N, Mennie J, Magos T, Stewart K. Desire for post bariatric body contouring in South East Scotland. J Plast Reconstr Aesthet Surg. 2013;66(1):87-94.

9. Mitchell JE, Crosby RD, Ertelt TW, et al. The desire for body contouring surgery after bariatric surgery. Obes Surg. 2008;18(10):1308-1312.

10. Gusenoff JA, Messing S, O'Malley W, Langstein HN. Temporal and demographic factors influencing the desire for plastic surgery after gastric bypass surgery. Plast Reconstr Surg. 2008;121(6):2120-2126.

**Complex loss of domain ventral hernias in initially Person with obesity: progressive pneumoperitoneum (PPP) and injection of botulinum toxin A (BTX) prior to surgical repair and abdominoplastic versus direct abdominal wall reconstruction: three case reports.**
ID: 142

Fabian Tycho Nehls[1], Martin Büsing[1], Vasiliki Christogianni[1]

[1]*Adipositaszentrum-Vest, Recklinghausen, Deutschland*

**Background:**
Worldwide increasing obesity and massive weight loss after bariatric surgery leads to a risk of huge ventral loss of domain hernias. To avoid an abdominal compartment syndrom after surgical repair, we conducted a two step approach**:** PPP and injection of BTX , followed by abdominal wall reconstruction using a mesh and remove of excess skin

**Materials and methods:**
From 2017-2019 we treated: First 46-year-old patient with an initial BMI 52 kg/m2 , initially underwent a Sleeve gastrectomy. 18 months a BMI of 27.9 kg/m2 and a huge ventral hernia, 26 cm in diameter. He couldn't lose more weight so we performed a laparotomie SADI bypass, reconstructed the abdominal wall with BIO-A 30x20cm mesh intraperitoneal, implanted in onlay technique a TIOMesh 30x30cm. We performed an abdominoplastic.
Second 49-year-old, with initial BMI of 42.5 and in ct domain ventral hernia of 17,4cm and a small 5,5cm hernia periumbilical. Patient rejected a bariatric operation, decided to change his lifestyle. 14 months later BMI of 36.1 kg/ m2. We injected BTX100U and insufflated for 5 days 3,1L CO2 as a progressive pneumoperitoneum (PPP). On day six we did a laparotomie and implanted two 20x30cm BIO-A-Mesh IPOM. In onlay technique we inserted two TIO2Mesh. We performed an abdominoplastic.
Third one 54 years old, initial BMI of 46.7, 8 month after sleeve gastrectomy BMI 32, showed a 15cm large ventral hernia.We injected BTX100U and insufflated for 5 days 3,1L CO2 as a PPP. We inserted in IPOM technique 2 BIO-A-Netzen 30x20cm and implanted 2 Progrip mesh 30x15cm epifascial and finished the reconstruction with an atypical abdominoplastic.

**Results:**
First patient without the conditioning developed an infection and an abdominal compartment syndrom and had to be treated with an abdominal Vacu-Seal System for 5 days. The wound healing was secondary. Second patient with conditioning had a postoperative intrabdominell pressure measurement which was normal and no problems. Third patient with conditioning developed a seroma and an infection which was treated with antibiotics and a subcutaneous Vacu-Seal System. No compartment

**Conclusion:**
Our cases suggest that a two step approach with injection of BTX and PPP followed by abdominal wall reconstruction and abdominoplastic lowers in former PwO the risk of an abdominal compartment after loss of domain hernia repair combined with remove of excess skin.

Abstracts – DCK 2024 – 141st Congress of the German Society of Surgery – Leipzig, April 24–26 • DOI 10.1515/iss-2024-2007
Innov Surg Sci 2024; 9 (Special Suppl 1): s280–s302

**Epidemiology and Treatment of Obstetric Brachial Plexus Injury in Germany: A National Cohort Study**
ID: 388

Tarik Alp Sargut[1], Kiarash Ferdowssian[1], Joan Alsolivany[1], Jörg Bahm[2], Nora F. Dengler[1]

[1]*Charité Campus Benjamin Franklin, Berlin, Deutschland*, [2]*Uniklinik RWTH Aachen, Aachen, Deutschland*

**Background:**
Obstetric brachial plexus injury (OBPI) is a serious and complex nerve injury in newborn children. Large-scale studies and guidelines are nowadays scarce and there are major regional differences in epidemiology and clinical management. This national cohort study aims to report on current epidemiology and therapeutic management strategies in Germany.

**Materials and methods:**
Patients hospitalized in Germany between January 1, 2005 and December 31, 2018 with OBPI as their primary diagnosis were identified using the 10th Revision of the International Classifcation of Disease (ICD-10) codes P14.0 (Erb paralysis), P14.1 (Klumpke paralysis) and P14.3 (Other brachial plexus birth injuries) alongside standardized operations and procedures codes (OPS).

**Results:**
A total of 2,069 patients with OBPI were included. Erb paralysis was the most frequent subtype. The number of total live births increased statistically significantly from 685,795 in 2005 to 787,523 in 2018 resulting in an overall OBPI rate of 0.21 per 1000 births over the years. The incidence of OBPI per 1000 live births decreased significantly by 46% from 0.28 in 2005 to 0.15 in 2018, displaying a mean annual decrease by 0.01 (± 0.025). Subgroup analysis also showed a significant decrease of all three ICD 10 codes (P14.0, P14.1 and P14.3). Simultaneously, the rates of caesarean delivery significantly increased ranging from 267.2 per 1000 live births in 2005 to 312.1 per 1000 live births in 2018.
The most frequent risk factor was large for gestational age (P08.0, P08.1) followed by cephalhaematoma (P12.0). Total numbers of surgical management ranged from 2-44% with a significantly increase between 2005 and 2018. Analysis of the different surgical treatment modalities showed an significant trend towards increasing numbers of surgical explorations with a decrease of nerve grafting.
Length of hospital stay among OBPI patients remained constant throughout the study period ranging from 1.0-6.0 days without significant change.

**Conclusion:**
OBPI is a rare disease with decreasing incidence in Germany paralleled by an increase of caesarean birth delivery rates between 2005-2018. Surgical management rates are rising. There is a significant trend towards increasing rates of microsurgical explorations and decreasing rates of nerve grafts. Large for gestational age was found to be the main risk factor.

Abstracts – DCK 2024 – 141st Congress of the German Society of Surgery – Leipzig, April 24–26 • DOI 10.1515/iss-2024-2007
Innov Surg Sci 2024; 9 (Special Suppl 1): s280–s302

**Supraclavicular first rib resection for thoracic outlet syndrome (TOS): correlation of patient rated outcome measures (PROMs) with length of posterior rib remnant**
ID: 464

Claudia Bauer[1]

[1]*Neurozentrum Oberaargau, Langenthal, Schweiz*

**Background:**
To assess the impact of length of posterior rib remnant on outcome of supraclavicular first rib resection for Thoracic Outlet Syndrome (TOS)

**Materials and methods:**
In a prospective study all patients undergoing supraclavicular first rib resection for TOS since 2016 have been enrolled. Patients had to complete Quick DASH (Disability of the Arm, Shoulder and Hand) and CBSQ (Cervical Brachial Symptom Questionnaire) before and 4 to 24 months after operation and report the percentage of improvement after operation. Length of rib remnant was measured on postoperative routine thorax x-ray from costovertebral joint to resection margin in mm.

**Results:**
108 supraclavicular first rib resections for TOS have been perfomed in 89 patients (21 % bilateral). 74 % were female, average age was 38 years (18-66). Patients rated the postoperative outcome as 79 % improvement. 13 patients were unable to rate the postoperative improvement in percent or were lost to follow up. Quick DASH scores showed an improvement of 65 % (66 preoperative vs. 23 postoperative) and CBSQ scores an improvement of 70 % (88 preoperative vs. 26 postoperative). Average length of rib remnant was 21 mm (8 to 38 mm). Analysis of subgroups revealed no correlation of length of rib remnant with postoperative Quick DASH or CBSQ scores respectively, but good correlation of self assessed improvement in percent and percentage of improvement of postoperative scores of Quick DASH and CBSQ.

**Conclusion:**
Quick DASH and especially CBSQ are reliable tools in evaluating outcome after surgery for TOS. Subgroup analysis revealed no correlation of length of posterior rib remnant with postoperative outcome. Supraclavicular first rib resection for TOS can offer consistently good long term results with high patient satisfaction in carefully selected patients.

Abstracts – DCK 2024 – 141st Congress of the German Society of Surgery – Leipzig, April 24–26 • DOI 10.1515/iss-2024-2007
Innov Surg Sci 2024; 9 (Special Suppl 1): s280–s302

**Health related quality of life and impact on work status in patients surgically treated for neurogenic thoracic outlet syndrome via a microsurgical supraclavicular approach with intraoperative neuromonitoring**
ID: 605

Joan Alsolivany[1], S Baruchi[1], Tarek Alp Sargut[1], Jennifer Reinisch[1], Anna Zdunczyk[1], Nora F Dengler[1]

[1]*Neurochirurgie, Charité Universitätsmedizin Berlin, Berlin, Deutschland*

**Background:**
Compression of the neurovascular bundle at the thoracic outlet with sensorimotor symptoms is referred to as neurogenic thoracic outlet syndrome (nTOS). The impact of microsurgical decompressive surgery via the supraclavicular approach under neuromonitoring has never been characterized in terms of health-related quality of life (QoL), and impact on individual work status.

**Materials and methods:**
Ethical approval was granted by local authorities (EA01/034/21). A validated questionnaire was sent to 18 surgically treated patients with nTOS between 2015 and 2023 Data is presented as median and [IQR].

**Results:**
The questionnaire was sent back by 88,9% of patients with a median age of 39,5 [31;45] years. Symptoms were present 3 years [2;8] before surgery. 13 patients were female, median age was 35 [31;44]. Before surgery, 41% of patients were absent of work, 17% were retired. Follow-up (FU) was accomplished at a median of 1,7 years [0,65;3,9] after surgery. 85% of patients reported a benefit from surgery. Pre-surgical VAS improved from 8 [8;8,4] to 4 [3;6] at FU ($p<0.001$). At FU, 23 % of patients were retired; 0 % due to nTOS. 82 % of patients returned back to work. Preoperative health related QoL on a scale from 0 to 100 was rated with a median of 60 [50;72,5] and was significantly improved at FU with a median of 85 [78;95] ($p<0.001$). Median dimensional values (1- no problems to 5 – not able to perform tasks) for mobility/self-care/activity/pain/ and anxiety improved from 3/3/4/3/3 to 2/2/2/2/1,5, respectively.

**Conclusion:**
A substantial number of patients had a benefit from surgery and significant QoL improvement with higher percentage of patients being part of the active workforce, after surgery. Health related QoL in patients treated by a microsurgical supraclavicular approach with intraoperative neuromonitoring was comparable to the German average population

**Transferability of cancer registry data to clinical practice in retroperitoneal sarcoma**
ID: 16

Franziska Neemann[1], Lina Jansen[2], Christian Silcher[1], Bernd Kasper[1], Peter Hohenberger[1], Dario Callegaro[3], Alessandro Gronchi[3], Marco Fiore[3], Rosalba Miceli[3], Frits van Coevorden[4], Winan van Houdt[4], Sylvie Bonvalot[5], Piotr Rutkowski[6], Jacek Skoczylas[6], Carol Swallow[7], Rebecca Gladdy[7], Dirk Strauss[8], Andrew Hayes[8], Chandrajit Raut[9], Mark Fairweather[9], Jens Jakob[1]

[1]*University Hospital Mannheim , Mannheim, Deutschland*, [2]*German Cancer Research Center (DKFZ), Epidemiological Cancer Registry Baden-Württemberg, Heidelberg, Deutschland*, [3]*Istituto Nazionale dei Tumori, Milano , Italy*, [4]*Netherlands Cancer Institute, Amsterdam, Netherlands*, [5]*Institut Curie , Paris, France*, [6]*Maria Sklodowska-Curie Memorial Cancer Center and Institute of Oncology, Warsaw, Poland*, [7]*Mount Sinai Hospital and Princess Margaret Cancer Centre University of Toronto, Toronto, Canada*, [8]*Royal Marsden Hospital NHS Foundation Trust, London, United Kingdom*, [9]*Dana-Farber Cancer Institute Harvard Medical School, Boston, United States*

**Background:**
Retroperitoneal sarcomas (RPS) are a rare, heterogeneous tumor group for which valid data is needed to guide treatment. To date, these come from cohorts on general soft tissue sarcomas. Cancer registries (CR) may be another possible source. It is questionable whether patients recorded in CR are comparable to an expert cohort in patient and tumor characteristics. This work compares CR and TARPS data to assess the representativeness of TARPS and the quality of CR data.

**Materials and methods:**
The TARPS cohort includes patients of primary RPS (years 2002 - 2011) and surgery in specialized centers (1). The CR Baden-Württemberg cohort includes all patients with primary RPS M0 (years 2016 - 2021, ICD-10 C.49.4/5, C48.x) and surgery within 12 months. To optimize the non-specific localizations (C48.x), only sarcoma-typical ICD O codes were chosen (see Onkozert certification). Patient, tumor and therapy factors were compared with Chi²-test and survival times were calculated with Kaplan Meier curves.

**Results:**
A total of 1007 (TARPS) and 319 (CR) patients were included. Patients in the CR cohort were significantly older (median: +9 years), had higher grading and a different histology distribution. The gender distribution, R status and proportion of patients with chemo- and radiotherapy were comparable. However, CR patients were less likely to have chemotherapy or radiotherapy before surgery. The 3-year survival probability was lower at 73% compared with 77% in CR.
The TARPS data are highly selected due to recruitment in specialized centers and have high data quality. CR data are population-based and thus representative of all RPS patients, but data quality is dependent on completeness and quality of reporting. Different codes for histological diagnosis make it difficult to compare cohorts.

**Conclusion:**
Overall, the quality of CR data appears sufficient to represent a cohort of RPS patients. It is important to build on this and optimize the existing reporting process for sarcomas to use the existing data from the state- and nationwide cancer registries to evaluate and develop evidence-based therapies in the futer.

(1) Gronchi A, Strauss DC, Miceli R, et al. Variability in Patterns of Recurrence After Resection of Primary Retroperitoneal Sarcoma (RPS): A Report on 1007 Patients From the Multi-institutional Collaborative RPS Working Group. *Ann Surg*. 2016;263(5):1002-1009. doi:10.1097/SLA.0000000000001447

Abstracts – DCK 2024 – 141st Congress of the German Society of Surgery – Leipzig, April 24–26 • DOI 10.1515/iss-2024-2007
Innov Surg Sci 2024; 9 (Special Suppl 1): s280–s302

**Contrast-enhanced ultrasonography guided core needle biopsy of soft tissue tumors: a retrospective study**
ID: 805

Anne-Christine Zygmunt[1], Florian Bösch[1], Markus Lerchbaumer[2], Ali Seif[3], Philipp Ströbel[3], Michael Ghadimi[1], Peter Jo[4]

[1]*University Medical Center Göttingen, Department for General, Visceral and Paediatric Surgery, Goettingen, Deutschland*, [2]*University Medical Center Charité Berlin, Department for Radiology and Paediatric Radiology, Berlin, Deutschland*, [3]*University Medical Center Göttingen, Department for Diagnostic and Interventional Radiology, Goettingen, Deutschland*, [4]*University Medical Center Göttingen, Department for Pathology, Goettingen, Deutschland*

**Background:**
In patients with soft tissue tumors (STT), treatment planning is largely dependent on tumor entity. Core needle biopsy (CNB) is the diagnostic standard in these cases. Ultrasonography (US) and in particular contrast-enhanced ultrasonography (CEUS) have provided very promising results in biopsy guidance. CEUS guided CNB in particular has been demonstrated to result in high sensitivity and specificity in relation to adequacy and accuracy of diagnosis. CEUS, allowing the visualization of micro-vasculature and areas of angiogenesis, may allow to perform the bioptic sampling at the most significant areas and therefore obtain highly accurate histological diagnosis of the tumor, in particular when dealing with large tumor dimensions and heterogenous areas in imaging, making it difficult to identify the most representative areas for sampling. Although a very useful imaging tool, CEUS is still not widely used in clinical practice, and only few reports have discussed its use in the evaluation of STT. Especially concerning abdominal or retroperitoneal STT there is a noticeable lack of data concerning CEUS guided CNB. The purpose of this study was to evaluate the role of CEUS in diagnosing STT.

**Materials and methods:**
This is a retrospective study of 40 patients subjected to CEUS-guided core needle biopsy to characterize STTs diagnosed from December 2021 to October 2023 at the sarcoma center of the University Medical Center Goettingen. US and CEUS were performed by the same two surgeons using the same ultrasound device (Logiq S8 General Electric machine XDClear, General Electric Healthcare, Wauwatosa, WI, USA), using an 8-µL/mL solution of sulfur hexafluoride microbubbles stabilized by a phospholipid shell (SonoVue, Bracco® SpA, Milan, Italy) as contrast medium. CEUS-guided bioptic sampling was carried out on each patient of tumoral areas enhanced by the contrast medium under local anesthesia using sterile conditions according to standard operating procedures with a sterile biopsy instrument (16 or 18G x 15 mm to 25 mm, CorVocet Biopsy System, Meritmedical, Sought Jordan, UT, USA). At least 2 to 3 core samples of tissue with an aspired length of 1,5 to 2,5 cm were obtained. No peri- or postinterventional complications occurred.

**Results:**
Of 19 males and 21 females, the masses biopsied were located in the abdomen (n=9), lower extremities (n=22), torso (n=7). The patients' ages ranged from 24 to 88 years (women age range 24 - 83; men age range 38 - 88). Re-biopsy was needed in only one malignant tumor due to insufficient amount of material for definite histopathological diagnosis. Accuracy of US-guided CNB after CEUS was determined by comparing the histology of the biopsy with the definitive diagnosis in 18 surgically resected samples (6 benign, 12 malignant). In resected samples histopathological diagnosis correlated with biopsy in 100%. In one case resection showed upgrade from atypic lipomatous tumor (ALT) to ALT with beginning progression to dedifferentiated liposarcoma G2. One case showed a downgrade in retroperitoneal liposarcoma from G2 to G1.

**Conclusion:**
In conclusion, CEUS-guided CNB is safe and (cost-)effective. CEUS, due to its ability to evaluate microvascular areas, has proven to be a promising method in guiding bioptic sampling of STT, directing the needle to the most significant and representative areas of the tumor. Sensitivity is extremely high in determining the histopathological diagnosis. Additionally, this study to our best knowledge evaluates the role of CEUS in STT located in the abdomen and retroperitoneum for the first time. Given the small number of patients evaluated in our study, it would be appropriate to obtain a larger sample size.

**Radiomics for the assessment of diffuse axonal injury (DAI) in patients with traumatic brain injury**
ID: 52

Anna-Katharina Meißner[1], Robin Gutsche[2], Enrico Budzejko[3], Christina Hamisch[1 3], Martin Kocher[4], Lenhard Pennig[5], Christian Nelles[5], Maximilian Ruge[4], Roland Goldbrunner[1], Philipp Lohmann[2]

[1]*Department of General Neurosurgery, Faculty of Medicine and University Hospital Cologne, University of Cologne, Cologne, Deutschland*, [2]*Inst. of Neuroscience and Medicine (INM-4), Research Center Juelich (FZJ), Juelich, Deutschland*, [3]*Department of Neurosurgery , Klinikum Fulda, Academic Hospital of the University of Marburg, Fulda*, [4]*Department of Stereotactic and Functional Neurosurgery, Faculty of Medicine and University Hospital Cologne, University of Cologne, Cologne*, [5]*Inst. for Diagnostic and Interventional Radiology, Faculty of Medicine and University Hospital Cologne, University of Cologne, Cologne*

**Background:**
De- and acceleration traumata can cause diffuse axonal injury (DAI) in 40-75% of patients with traumatic brain injury (TBI) which is associated with a high risk of long-term morbidity. The diagnosis of DAI on CT is challenging due to the lack of structural abnormalities. To overcome this limitation, MR imaging including diffusion- and susceptibility-weighted sequences is necessary, but not always suitable for instable ICU patients. Therefore, DAI remains underdiagnosed. Radiomics, a method from the field of artificial intelligence, not established in DAI patients yet, offers the opportunity to extract additional diagnostic information from routine imaging data. The purpose of this work was the evaluation of the potential of multimodal radiomics for an improved diagnosis of DAI.

**Materials and methods:**
MR imaging including T2, FLAIR, DWI and SWI/T2* sequences was performed in 42 patients suspicious of DAI due to the clinical state, and a control group (n=44). All patients received a trauma CT scan at the time of admission. DAI was diagnosed by an experienced neuroradiologists based on MR imaging data. To define the target volume for radiomics feature extraction, an MRI-based atlas of the predilection areas for DAI was developed. After radiomics feature extraction based on MRI as well as CT data, a test-retest analysis was performed to identify robust features prior to feature selection. The radiomics model was trained and validated by five-fold cross validation. Diagnostic performance was evaluated using receiver operating characteristic (ROC) analyses.

**Results:**
The MRI radiomics features showed significant differences between patients with DAI and healthy controls, especially in the thalamus, basal ganglia, and corpus callosum. The developed random forest classifier using the MRI radiomics signature yielded an area under the ROC curve (AUC) of 0.89, 0.86 and 0.95 in these areas. Further, the CT radiomics features also showed significant differences between patients and healthy controls. The developed CT radiomics signature yielded an AUC of 0.85, 0.85, and 0.80 for the respective brain regions.

**Conclusion:**
MRI as well as CT based radiomics analysis appears feasible for the assessment of DAI at an early stage. Especially a radiomics classifier based on CT is of high clinical value as a screening tool for patients with severe and mild TBI. Further evaluation of the developed models in an external test data set is currently ongoing.

**Patient-specific 3D-printed helmet after craniectomy: A technical note**
ID: 298

Johannes Wach[1], Martin Vychopen[1], Dirk Winkler[1], Erdem Güresir[1], Fabian Kropla[1], Ronny Grunert[1]

[1]*Klinik und Poliklinik für Neurochirurgie Universitätsklinikum Leipzig, Leipzig, Deutschland*

**Background:**
Decompressive hemicraniectomy (DH) is a life-saving surgery for malignant brain infarctions, intracerebral hemorrhages, and severe traumatic brain injuries (TBI) (1, 2). It involves removing a significant part of the skull and opening the dura mater (3). To reduce brain swelling and intracerebral pressure, the scalp is closed without reimplanting the bone. DH is especially crucial within 48 hours for lowering mortality, particularly in young patients with malignant middle cerebral artery infarctions (4). However, DH can lead to complications like wound healing problems, infections, brain herniation, subdural fluid collections, seizures, and hydrocephalus (5, 6), with complication rates ranging from 22% to 54% in literature (7).
Following DH and sufficient recovery, patients undergo autologous cranioplasty, delaying it for over three months, especially in cases involving hydrocephalus, seizures, and wound complications, is recommended (8). This approach seems better for potential recovery from motor or cognitive deficits compared to early cranioplasty (9). However, the risk of falls and injuries is higher in the early weeks post-DH due to motor and coordination limitations. Currently, there is inadequate data and recommendations for optimal care of skull defects after DH (10). Innovative methods using methyl methacrylate bone cement are explored as alternatives to heavy, rigid, industrially available helmets (11).
Using 3D technology offers the opportunity to create customized external skull prostheses or helmets. Postoperative CT scans don't consider gravity-induced brain parenchyma shifts in mobile hemicraniectomy patients. Therefore, a 3D camera scanning technology-based manufacturing process was developed for patient-specific helmets, potentially reducing hospital stays and associated costs, encouraging early mobilization, and alleviating patients' injury fears (12, 13). Customized 3D-printed helmets show promise for improving patient care and reducing hospitalization duration, possibly easing the healthcare system's economic burden.

**Materials and methods:**
Once wound healing is complete and patients display sufficient cognitive ability, readiness for outpatient monitoring, and mobility, postoperative scannings are performed using the handheld 3D structured light scanner Artec Leo (Artec 3D, Luxembourg). Figure 1 shows the timepoint of the production process in the clinical workflow. Artec Studio V15 software (Artec 3D, Luxembourg) processes the scan data, creating a 3D model. Scanning takes about 10 minutes, and post-scan processing in the software takes 30 minutes. Autodesk's Fusion 360 software (California, USA) is used for the design and individual production of the 3D-printed helmet. Unlike industrial manufacturing, this process involves patient-specific customization, including grid structures and perforations using nTopology software (New York, USA). The 3D printer HP Multi Jet 5200 (California, USA) is used for printing, employing a powder-based method (Polyamide 12) and a subsequent 6-8-hour cooling period. A glass bead blasting machine removes excess powder from the helmet. Finally, institutional orthopedic technicians line the helmet with padding and closures.

**Results:**
Figure 2 shows an individualized helmet produced using 3D printing for a 74-year-old male patient who underwent decompressive hemicraniectomy due to a severe acute subdural hematoma following a severe TBI. The helmet fit perfectly, and the patient complied with its daily use. The helmet's design did not interfere with wound healing, and no adverse events like itching, decubitus, or skin irritations occurred. The helmet remained intact until autologous cranioplasty.

**Conclusion:**

3D printing is a viable method for creating individualized patient-specific helmets for severe TBI patients who have undergone DH. Further research is needed to enable institutional production by neurosurgeons and implement a fast-track mobilization program for patients.

**References:**

1. Hutchinson PJ, Adams H, Mohan M, Devi BI, et al.. Decompressive Craniectomy versus Craniotomy for Acute Subdural Hematoma. N Engl J Med. 2023 Jun 15;388(24):2219-2229. doi: 10.1056/NEJMoa2214172.
2. Neugebauer H, Fiss I, Pinczolits A, et al. Large Size Hemicraniectomy Reduces Early Herniation in Malignant Middle Cerebral Artery Infarction. Cerebrovasc Dis. 2016;41(5-6):283-90. doi: 10.1159/000443935.
3. Güresir E, Vatter H, Schuss P, et al. Rapid closure technique in decompressive craniectomy. J Neurosurg. 2011 Apr;114(4):954-60. doi: 10.3171/2009.12.JNS091065.
4. Vahedi K, Hofmeijer J, Juettler E, et al. DECIMAL, DESTINY, and HAMLET investigators. Early decompressive surgery in malignant infarction of the middle cerebral artery: a pooled analysis of three randomised controlled trials. Lancet Neurol. 2007 Mar;6(3):215-22. doi: 10.1016/S1474-4422(07)70036-4.
5. Schuss P, Borger V, Güresir Á, et al. Cranioplasty and Ventriculoperitoneal Shunt Placement after Decompressive Craniectomy: Staged Surgery Is Associated with Fewer Postoperative Complications. World Neurosurg. 2015 Oct;84(4):1051-4. doi: 10.1016/j.wneu.2015.05.066.
6. Berghmans M, de Ghellinck L, De Greef J, et al. Outcome of Patients with Surgical Site Infection after Craniotomy. Surg Infect (Larchmt). 2022 May;23(4):388-393. doi: 10.1089/sur.2021.260.
7. Stiver SI. Complications of decompressive craniectomy for traumatic brain injury. Neurosurg Focus. 2009 Jun;26(6):E7. doi: 10.3171/2009.4.FOCUS0965.
8. Borger V, Schuss P, Kinfe TM, et al. Decompressive Craniectomy for Stroke: Early Cranioplasty Is a Predictor for Postoperative Complications. World Neurosurg. 2016 Aug;92:83-88. doi: 10.1016/j.wneu.2016.04.113.
9. De Cola MC, Corallo F, Pria D, et al. Timing for cranioplasty to improve neurological outcome: A systematic review. Brain Behav. 2018 Nov;8(11):e01106. doi: 10.1002/brb3.1106.
10. Honeybul S. Decompressive craniectomy: a new complication. J Clin Neurosci. 2009 May;16(5):727-9. doi: 10.1016/j.jocn.2008.06.015.
11. Boström S, Bobinski L, Zsigmond P, et al. Improved brain protection at decompressive craniectomy--a new method using Palacos R-40 (methylmethacrylate). Acta Neurochir (Wien). 2005 Mar;147(3):279-81; discussion 281. doi: 10.1007/s00701-004-0480-4.
12. Andruszkow H, Deniz E, Urner J, et al. Physical and psychological long-term outcome after traumatic brain injury in children and adult patients. Health Qual Life Outcomes. 2014 Feb 26;12:26. doi: 10.1186/1477-7525-12-26.
13. Clark DE, Lowman JD, Griffin RL, et al. Effectiveness of an early mobilization protocol in a trauma and burns intensive care unit: a retrospective cohort study. Phys Ther. 2013 Feb;93(2):186-96. doi: 10.2522/ptj.20110417.

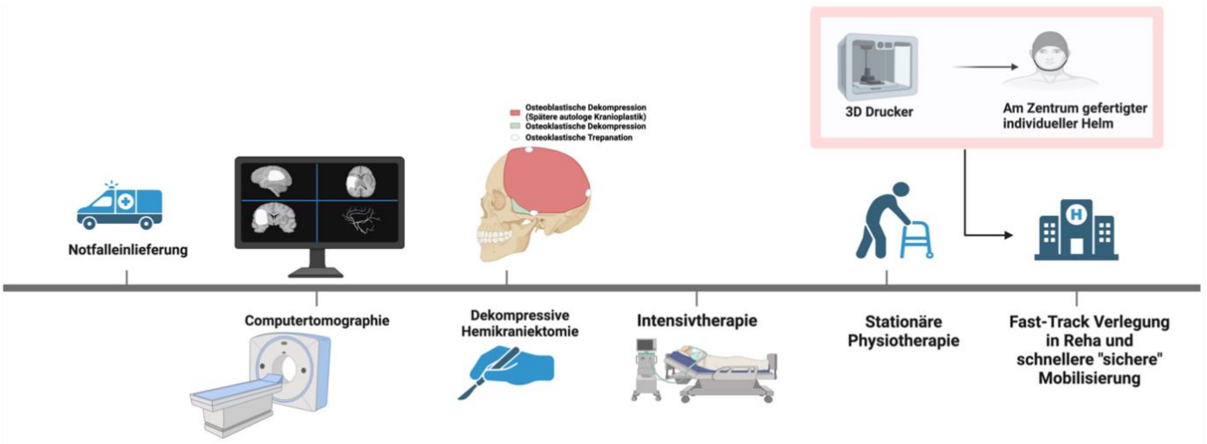

**Figure 1**

Abstracts – DCK 2024 – 141st Congress of the German Society of Surgery – Leipzig, April 24–26 • DOI 10.1515/iss-2024-2007
Innov Surg Sci 2024; 9 (Special Suppl 1): s280–s302

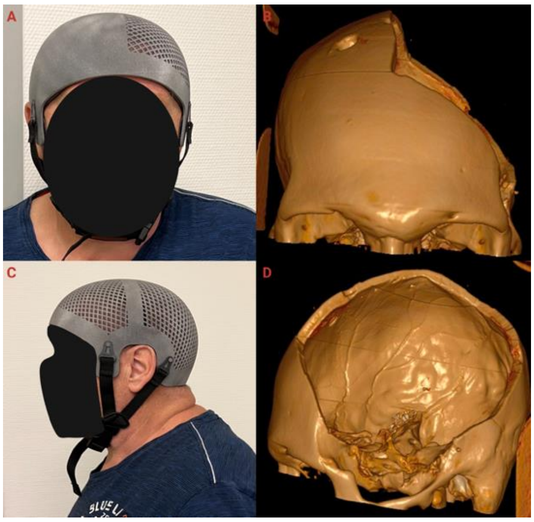

**Figure 2**

Abstracts – DCK 2024 – 141st Congress of the German Society of Surgery – Leipzig, April 24–26 • DOI 10.1515/iss-2024-2007
Innov Surg Sci 2024; 9 (Special Suppl 1): s280–s302

**Clinical management of 2697 mild TBI patients presenting to a neurosurgical emergency department**
ID: 726

Tamara Schink[1], Shyrete Bahtijari[1], Leon Kromer[1], Andreas Unterberg[1], Sandro Krieg[1], Alexander Younsi[1]

[1]*Universitätsklinikum Heidelberg, Neurochirurgie, Heidelberg, Deutschland*

**Background:**
Mild traumatic brain injury (TBI) is an increasing phenomenon, partially associated with neurological impairment and reduced quality of life. Given its socioeconomic relevance, assessing whether patients require neuroimaging, monitoring or interventions is becoming increasingly important. We, therefore, aimed to describe clinical management in mild TBI patients presenting to a neurosurgical emergency department (ED).

**Materials and methods:**
All mild TBI patients (GCS 13-15) admitted to the ED of a neurosurgical maximum care provider in Germany were prospectively assessed between April 2021 and October 2023. Demographic and clinical variables were collected and the necessity for neuroimaging, admission to the regular ward/ICU and medical or surgical interventions were analyzed.

**Results:**
Over a period of 30 months, 2697 mild TBI patients could be included (51% male; mean age 51± 24 years). The most common cause of injury was an incidental fall (n=1663; 62%), typically occurring in the home environment. Neurological deficits were rare (n=113; 4,2%), but 389 patients (14%) suffered from amnesia and 302 (11%) had experienced vomiting. Antithrombotic medication was taken by 23% of patients (n=619) and 7% (n=199) were under the influence of alcohol. Brain computed tomography (CT) imaging was performed in 69% of cases (n=1856). Intracranial pathologies were observed in 472 patients (25%). Medical or surgical interventions were performed in 1104 patients (41%), including, inter alia, i.v.-analgesia (n=736; 67%), antibiotic treatment (n=117; 11%) and neurosurgery (n=77; 7%). Admission to the normal ward or ICU was deemed necessary in 17% of cases (n=456) with “pathological CT finding” being the most common indication (n=347; 76%) and the mean length of hospital stay being 4 ± 4 days.

**Conclusion:**
Mild TBI is associated with neuroimaging, medical/surgical interventions, and hospital admission in a substantial number of patients presenting to a neurosurgical maximum care provider. Our findings suggest that the causal injuries might be less “mild” than suspected and should raise awareness for their adequate clinical management.

Abstracts – DCK 2024 – 141st Congress of the German Society of Surgery – Leipzig, April 24–26 • DOI 10.1515/iss-2024-2007
Innov Surg Sci 2024; 9 (Special Suppl 1): s280–s302

**Care paths, clinical management and decision making in 520 mild TBI patients over the age of 80 presenting to a neurosurgical emergency department**
ID: 729

Tamara Schink[1], Shyrete Bahtijari[1], Andreas Unterberg[1], Sandro Krieg[1], Alexander Younsi[1]

[1]*Universitätsklinikum Heidelberg, Neurochirurgie, Heidelberg, Deutschland*

**Background:**
Mild traumatic brain injury (TBI) is an increasing phenomenon, partially associated with neurological impairment and reduced quality of life. Given the current demographic change, assessing possible factors associated with an unfavorable outcome after mTBI in the elderly is becoming increasingly important. We, therefore, aimed to describe the clinical management and outcome of elderly mTBI patients presenting to a neurosurgical emergency department (ED).

**Materials and methods:**
All TBI patients admitted to the ED of neurosurgical maximum care provider in Germany were prospectively assessed between April 2021 and October 2023. Demographic and clinical variables were collected and the necessity for neuroimaging, admission to the regular ward or ICU, medical or surgical interventions and the clinical outcome at discharge (mRS) were analyzed.

**Results:**
Over a period of 30 months, 2883 patients were assessed, of which 2697 presented with mTBI (GCS 13-15). For further analysis, only the 520 (19%) mTBI patients aged ≥80 years (44% male; mean age 86 ± 4,3 years) were included. The most common cause of injury was an incidental fall (n=497; 96%), typically occurring in the home environment. Neurological deficits were rare (n=32; 6%), but 66 patients (13%) suffered from amnesia and 35 (7%) had experienced vomiting. Antithrombotic medication was taken by 66% of patients (n=342) and only 1,5% (n=8) were under the influence of alcohol. Intracranial pathologies on brain CT imaging were observed in 186 patients (36%). Medical or surgical interventions were performed in 289 patients (56%), including i.v.-analgesia (n=216; 75%), superficial wound care (n=190; 66%) and neurosurgery (n=28; 10%). Admission to the hospital was deemed necessary in 32% of patients (n=167), from which 52 (31%) were admitted directly to the ICU. The mean length of hospital stay was 4 ± 4 days and 70 (42%) of patients had a mRS of 0 or 1 at discharge. Unsurprisingly, nonagenarians had significantly higher Odds of having a worse outcome (measured by mRS) in comparison to octagenarians (OR 3.1, 95% CI 1.91 to 4.88, $p<0.05$).

**Conclusion:**
Our study highlights a clear need for better-focused care strategies for elderly patients experiencing mild TBI, especially given the commonality of falls and observed imaging findings. At the same time, a high rate of hospital and even ICU admissions is alarming. With older patients, particularly those in their 90s, showing worse outcomes, it's crucial to continue research to improve care and outcomes for this vulnerable group after an injury.
